# Hospital News

**Published:** 1922-09

**Authors:** 


### Hospital News.

The new out-patients' department has been opened at Gateshead Children's Hospital.

The Bakewell and District War Memorial Cottage Hospital has been opened, free of debt, by the Duke of Devonshire.

On August 21 the new out-patients' department of the Royal Hospital for Women and Children, in Waterloo Road, was opened.

Princess Mary has consented to open the new children's ward, which was the city's wedding gift to her, at the Leeds General Infirmary, on October 2.

The Whickham and District War Memorial Hospital was opened on August 26. Mr. F. W. Morrision is chairman of the Committee of Management.

The extensions decided on at the Beckenham Cottage Hospital will raise the number of beds from 25 to 40. Mr. W. A. Pite, F.R.I.B.A., is the architect.

A new institution known as the Abertillery and District Hospital is being opened at Aberbeeg. Mr. T. H. Mytton is chairman of the Hospital Committee.

The foundation stone has been laid of a new hospital and dispensary at Retford. The institution will form the second part of the War Memorial of the town. There will be nine wards.

A site for a new hospital, overlooking the river, is being bought by the Teignmouth Hospital Committee in a field on the Barnfield estate. Plans and some funds are in hand, but building will be postponed till prices drop further.

The proposed extension of the Royal Northern Hospital, to form part of the Islington War Memorial, is to be begun shortly. The architects are Messrs. Adams and Holden. The extension is to be a casualty department with observation wards.

An amalgamation between the Herefordshire General Hospital and the Victoria Eye and Ear Hospital, Hereford, is being discussed, and will probably be carried through if the entity of the latter can be preserved, as has lately been done, for example, at Norwich.

The opening of the new annexe for the night nurses at York County Hospital enables almost all the staff to be housed upon the premises. The annexe contains 14 rooms. The Home is dedicated to the memory of Lady Elizabeth Hastings, one of the founders of the hospital.

The Royal Albert Institution for the Feeble-minded, Lancaster, which claims to have been a pioneer in the milking of cows by electricity, by a wireless installation lately enabled the patients to enjoy a concert at The Hague. It is hoped to make these transmitted concerts a regular part of the patients' recreation.

Serious charges have been made against the condition of certain wards in St. Andrew's Hospital, Bromley, E., which are described as verminous in the report of the Poplar and Stepney Sick Asylums Board. It is stated that Wards E and F have been found covered with bugs, that the floors are in a shocking condition, and that the wards must be stripped and dismantled if a thorough cleansing is to be made. The Labour members on the Poplar Board of Guardians have called attention to the matter. The beds in the tainted wards have been stoved, and the removal of the patients to other wards has created over-crowding. The chairman of the Visiting Committee, Mr. Partridge, has stated that the present deplorable condition is due to the lack of supervision on the part of the old board. It is also contended that the nurses are overworked and badly accommodated, too few in number and on duty for too long hours. Excuses, of course, have been made, but they do not alter the facts that in every modern infirmary patients are properly cleansed on arrival, and that an infested institution is a condemned institution for hospital purposes.

